# Is Altered Central Pain Processing Related to Disease Stage in Chronic Pancreatitis Patients with Pain? An Exploratory Study

**DOI:** 10.1371/journal.pone.0055460

**Published:** 2013-02-06

**Authors:** Stefan A. W. Bouwense, Søren S. Olesen, Asbjørn M. Drewes, Jens B. Frøkjær, Harry van Goor, Oliver H. G. Wilder-Smith

**Affiliations:** 1 Pain and Nociception Neuroscience Research Group, Department of Surgery Radboud University Nijmegen Medical Center, Nijmegen, The Netherlands; 2 Mech-Sense, Department of Gastroenterology and Department of Radiology, Aalborg Hospital, Aarhus University Hospital, Aalborg, Denmark; 3 Center for Sensory-Motor Interaction (SMI), Department of Health Science and Technology, Aalborg University, Aalborg, Denmark; 4 Department of Anaesthesiology, Pain and Palliative Care, Radboud University Nijmegen Medical Center, Nijmegen, The Netherlands; University of South Australia, Australia

## Abstract

**Background:**

The most dominant feature in chronic pancreatitis is intense abdominal pain. Changes in spinal and/or supraspinal central nervous system pain processing due to visceral nociceptive input play an important role in this pain. How altered pain processing is related to disease stage still needs study.

**Methodology/Principal Findings:**

Sixty chronic pancreatitis patients were compared to 15 healthy controls. Two subgroups of pancreatitis patients were defined based on the M-ANNHEIM severity index of chronic pancreatitis; i.e. moderate and severe. Pain detection and tolerance thresholds for pressure and electric stimuli were measured in six selected dermatomes (C5, T4, T10, L1, L4 and T10BACK). In addition, the conditioned pain modulation response to cold pressor task was determined. These measures were compared between the healthy controls and chronic pancreatitis patients. Severe pancreatitis patients showed lower pain thresholds than moderate pancreatitis patients or healthy volunteers. Healthy controls showed a significantly larger conditioned pain modulation response compared to all chronic pancreatitis patients taken together.

**Conclusions/Significance:**

The present study confirms that chronic pancreatitis patients show signs of altered central processing of nociception compared to healthy controls. The study further suggests that these changes, i.e. central sensitization, may be influenced by disease stage. These findings underline the need to take altered central pain processing into account when managing the pain of chronic pancreatitis.

## Introduction

Intense upper abdominal pain is common in chronic pancreatitis patients and is the most important predictor of health-related quality of life. [1], [2] The etiology of pain remains to be elucidated and no generally accepted guidelines exist for its treatment. Initial treatment typically consists of a low fat diet and non-narcotic analgesics. [2] Alternatives to medical treatment, e.g. pancreatic surgery, thoracoscopic splanchnicectomy and lithotripsy, may have an effect on pain in selected patients. [3]–[5] If no acceptable pain relief is obtained, opioids remain the mainstay for the management of pain. However, opioids have many adverse effects such as negative influence on gastrointestinal motility, central nervous system toxicity, addiction and abuse potential, and sometimes opioid induced hyperalgesia. [6] Thus new treatment regimes for the debilitating pain of chronic pancreatitis are still needed. Adjuvant therapy with, e.g., pregabalin has recently been proven to be effective in chronic pancreatitis. [7], [8] However, centrally acting agents i.e. gabapentinoids or tricyclic antidepressants are not yet an accepted part of pain treatment in chronic pancreatitis.

Answering questions like “How do chronic pancreatitis patients process pain and how does altered pain processing relate to their pain experience?” is fundamental for the design of new therapeutic strategies. In human experimental pain models basic pain mechanisms can be explored by quantitative sensory testing (QST), electroencephalography or MRI. [9]–[12] These techniques provide insight into various aspects of pain processing during progression of a painful disease, as well as before and after a therapeutic intervention [13].

QST is increasingly used to study pain mechanisms in painful conditions. [13]–[16] A key described alteration in chronic pancreatitis patients is segmental and generalised hyperalgesia, often present despite (or because of) opioid usage. [9], [17] These changes are similar to those seen in neuropathic pain syndromes. [11] Their presence suggests that increased sensitivity in the central nervous system at spinal and/or supraspinal sites (“central sensitization”) plays an important role in chronic pancreatitis pain, and that this is not effectively modulated by current opioid based therapies. [11] Conditioned pain modulation (CPM) is a dynamic QST paradigm designed to activate and measure pain modulating mechanisms, e.g. via descending inhibitory control where brain stem centers act on nociceptive neurons in the dorsal horn of the spinal cord. [18] An impaired CPM response has been reported in chronic pancreatitis as well as in other gastrointestinal diseases and neuropathic conditions exhibiting hyperalgesia [19]–[21].

At present, comprehensive comparisons between chronic pancreatitis patients at different disease stages and compared with a healthy population regarding pain processing are not available. Such observations related to disease progression may also be of clinical value for other chronic painful disorders e.g. ulcerative colitis and Crohn’s disease [22].

The objective of this study is to investigate the difference in pain sensitivity and modulation between healthy subjects and chronic pancreatitis patients using QST to determine 1) pressure pain thresholds, 2) electric pain thresholds and 3) CPM response. Our hypothesis is that pain in chronic pancreatitis is accompanied by alterations in pain processing, and that this is influenced by disease stage [23].

## Methods

### Study Patients

The study was approved by the responsible Ethical Committees in both countries (CMO region Arnhem-Nijmegen, Nijmegen, The Netherlands and The local Ethics Committee North Region, Aalborg, Denmark) and all patients provided written informed consent. Patients were recruited for an investigator initiated double-blind, placebo-controlled, parallel-group study of increasing doses of pregabalin conducted in the Netherlands (department of Surgery, Radboud University Nijmegen Medical Center) and Denmark (department of Gastroenterology, Aalborg Hospital, Aarhus University Hospital. The study was powered on a clinical primary outcome measure which is presented in another manuscript [7] and not on the QST measurements that are described in this manuscript. The present study only presents the baseline QST results of all the 64 patients that were included in this trial.

To be included in this study, patients needed to have chronic abdominal pain typical for pancreatitis (i.e. dull epigastric pain more than 3 days per week for at least 3 months) and a diagnosis of chronic pancreatitis based on The Mayo Clinic diagnostic criteria. [24] Patients were excluded from the study if they had a painful condition other than chronic pancreatitis, an active (or history of) major depression, severe renal impairment, an abnormal electrocardiogram at screening, allergy to pregabalin or any of it components and were pregnant or lactating.

### Healthy Controls

A healthy control group was recruited in Denmark for comparison with our chronic pancreatitis group. The controls did not have any active disease and no history of a medical condition that could interfere with our pain measurements. Measurements were performed in females in the same phase of the menstrual cycle. Informed consent was provided by all healthy controls.

### Quantitative Sensory Testing

QST took place using a standard temporal test sequence. [9] Testing in females with pancreatitis was not standardized with regard to phase of the menstrual cycle because all pancreatitis patients had amenorrhea or were postmenopausal. After initial QST training, pressure pain thresholds were obtained for muscles overlying bone using a pressure algometer with a 1.0-cm^2^ probe (Somedic Sales AB, Horby, Sweden), at each of the following sites on the dominant body side: lower neck (C5 dermatome), sternum (T4 dermatome), pancreatic site (dorsal (T10 dermatome) and ventral (T10BACK dermatome)), hip region (L1 dermatome) and knee (L4 dermatome).

The pancreatic and more distant dermatomes were chosen to permit observation of segmental and spreading hyperalgesia respectively. The upper abdominal area (T10 ventral and dorsal) was chosen to detect segmental hyperalgesia because dorsal horn neurons receiving painful stimuli from this skin area also receive nociceptive stimuli from the pancreas (i.e. pancreatic area). To examine spreading and generalized hyperalgesia we chose 2 dermatomes (proximal and dorsal) near the pancreatic area (dermatomes T4 and L1) and 2 dermatomes more distant (proximal and dorsal) from the pancreatic area (dermatomes C5 and L4). The more distant areas were chosen to act as a control area likely unaffected by pancreatic nociceptive input because the nociceptive pathways from these areas are well-separated from those coming from the pancreas at both peripheral and spinal levels.

Two thresholds were measured: pressure pain detection threshold (pPDT) and pressure pain tolerance threshold (pPTT).

Thresholds to electric constant current skin stimulation (Digistim; Biometer A/S, Copenhagen, Denmark; tetanic stimulation at 100 Hz, 0.2-ms square waves, self-adhesive electrodes 3 cm apart) were measured on the same sites as for pressure stimulation. Two thresholds were measured: electric pain detection threshold (ePDT) and electric pain tolerance threshold (ePTT).

The conditioned pain modulation (CPM, previously known as DNIC) paradigm was carried out to test the ability of the patient to generate descending inhibitory modulation. [25], [26] Thus pressure pain tolerance thresholds (pPTT, the test stimulus) were determined before and after the cold pressor task (the conditioning stimulus), and the CPM effect was determined as the relative change (%) in pPTT. For the cold pressor task the dominant hand was immersed in ice-chilled water (1.0°C±0.3°C) continuously stirred by a pump. The patient was told to remove the hand from the water after 2 minutes of immersion - or sooner if the pain was considered to be intolerable – and the immersion time noted. Immediately after the cold pressor task, the subjects rated the pain experienced during the test by use of a visual analogue scale for quality control purposes. pPTT were obtained in the non-dominant L4 dermatome (knee) immediately before and after ice-water immersion.

### Disease Stage

We formed two groups of patients based on ‘the M-ANNHEIM severity index of chronic pancreatitis’ which is a validated clinical disease stage classification for chronic pancreatitis. [27] The M-ANNHEIM classification system incorporates etiology, different stages of the disease, and various degrees of clinical severity. The M-ANNHEIM scoring system for a clinical severity index is a simple, accurate and noninvasive tool in clinical practice and may be helpful in investigating the impact and interaction of various risk factors on the course of the disease. Clinical severity is based on pain control, surgical interventions, pancreatic endocrine and exocrine insufficiency, morphological status and severe organ complications.

We divided the patients in two groups based on their score, namely: ≤10 points, moderate chronic pancreatitis group (including: minor and increased severity level) and >10 points, severe chronic pancreatitis group (including: advanced and marked severity level).

### Statistical Analysis

The study was powered to detect a difference in average daily pain scores of 25% between groups during the 3 weeks of study treatment. We determined that a study with 30 patients per group was needed to provide a power of 90% with the use of a 2-sided significance level of 0.05. Hence, the sample size was set at 64 patients to allow for possible dropouts.

We performed statistical analysis using the Statistica for Windows Software Package (Release 6.0, Statsoft Inc., Tulsa, OK, USA). All results are given as means with standard deviations or 95% confidence intervals as appropriate. We analyzed QST results using a mixed model two-way ANOVA with one between subjects factor (GROUP; i.e. a healthy control group and two chronic pancreatitis groups according to disease stage) and one within subjects factor (SITE; consisting of the six dermatomes mentioned under quantitative sensory testing). We analyzed CPM results using a one-way ANOVA with one between subjects factors (GROUP; as above). Post hoc analysis was performed using Fisher’s least significant difference test.

## Results

### Study Population

**Figure 1:**
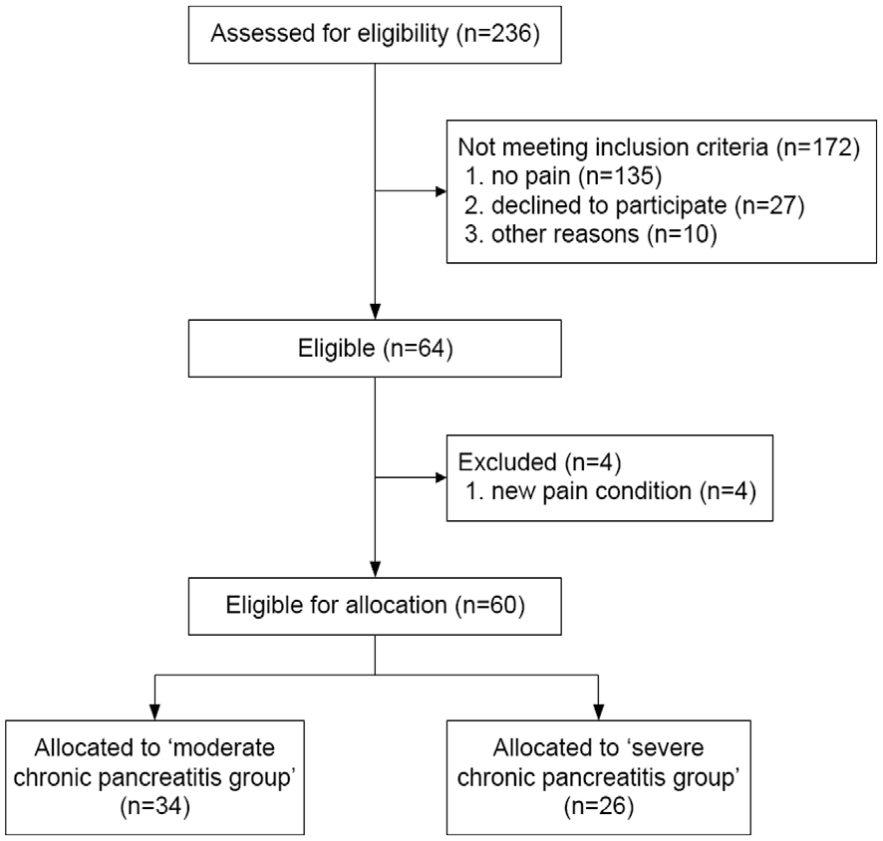
Study enrollment and randomization. The majority of patients ‘not meeting inclusion criteria’ had either died, was pain free or was no longer being treated in either of the hospitals.

From October 2008 to May 2010 a total of 236 patients diagnosed with chronic pancreatitis in the last five years in one of both hospitals were screened and 64 patients were randomized; the study was completed without any incident. The majority of patients not meeting inclusion criteria were pain free, had passed away or were no longer being treated in either of the hospitals. From those 64 patients, four patients were excluded due to a new pain condition (e.g. complication of chronic pancreatitis requiring surgery, tooth-abscess, emergency vascular surgery and diagnosis of IBD) that would interfere with their QST measurements (Figure 1). All patients in this per protocol analysis (24 women, 36 men; mean age 54±11) had pain due to chronic pancreatitis and were on a stable analgesic therapy. The healthy control group consisted of 15 volunteers. The moderate chronic pancreatitis group consisted of 34 patients and the severe group of 26 patients (Table 1). Pancreatitis groups were statistically comparable except – as expected – for previous interventions. More demographic data on the study population and control group are listed in Table 2.

**Table 1:** M-ANNHEIM severity index of chronic pancreatitis and distribution of patients.

	Severity level	Point range	Frequency no. (%)
M-ANNHEIM A	Minor	0–5	4 (7)
M-ANNHEIM B	Increased	6–10	30 (50)
M-ANNHEIM C	Advanced	11–15	21 (35)
M-ANNHEIM D	Marked	16–20	4 (7)
M-ANNHEIM E	Exacerbated	>20	1 (2)

M-ANNHEIM scoring system points are added together, and the sum is used to categorize a patient’s disease according to the M-ANNHEIM severity index.

**Table 2:** Demographic and clinical characteristics of patients and healthy controls.

	Healthy controls (n = 15)	Moderate chronicpancreatitis group (n = 34)	Severe chronic pancreatitis group (n = 26)
Age (years)	40±9*	53±11	53±11
Males - no. (%)	8 (53)	24 (71)	12 (46)
Etiology - no. (%)			
- Toxic-metabolic	0	17 (50)	13 (50)
- Idiopathic	0	13 (38)	8 (31)
- Genetic	0	1 (3)	1 4)
- Autoimmune	0	0	1 (4)
- Recurrent and severe acute pancreatitis	0	2 (6)	1 (4)
- Obstructive	0	1 (3)	2 (8)
Diary pain score (numeric rating score 0–10)			
- Average pain	0	4±2	4±2
- Maximal pain	0	5±2	6±2
Concomitant analgesics - no. (%)†			
- None	0	4 (12)	1 (4)
- Weak analgesics	0	7 (21)	10 (39)
- Strong analgesics	0	23 (68)	15 (58)
MEQ/day	0	112±132	72±71
Antidepressants - no. (%)	0	6 (18)	6 (23)
Duration of chronic pancreatitis (months)	0	113±85	100±75
Diabetes mellitus - no. (%)	0	4 (12)	14 (54)*
Previous interventions for chronic pancreatitis – no. (%)	0	2 (6)	13 (50)*
- Pancreas resection/drainage procedures	0	2 (6)	8 (31)*
- Thoracoscopic splanchnic denervation	0	1 (3)	7 (27)*
- Coeliacus blocade	0	0	2 (8)*
Patients treated with enzymes for pancreatic exocrineinsufficiency - no. (%)	0	11 (32)	17 (65)

All values are means with standard deviations unless mentioned otherwise. Percentages may not total 100 due to rounding. †Weak analgesics were defined as NSAIDS, paracetamol, codeine and tramadol. Strong analgesics were defined as opioid based therapies. ‘MEQ’ is morphine equivalents. Values marked with an asterisk were significantly different from each other.

### Thresholds to Pressure Stimulation

**Figure 2:**
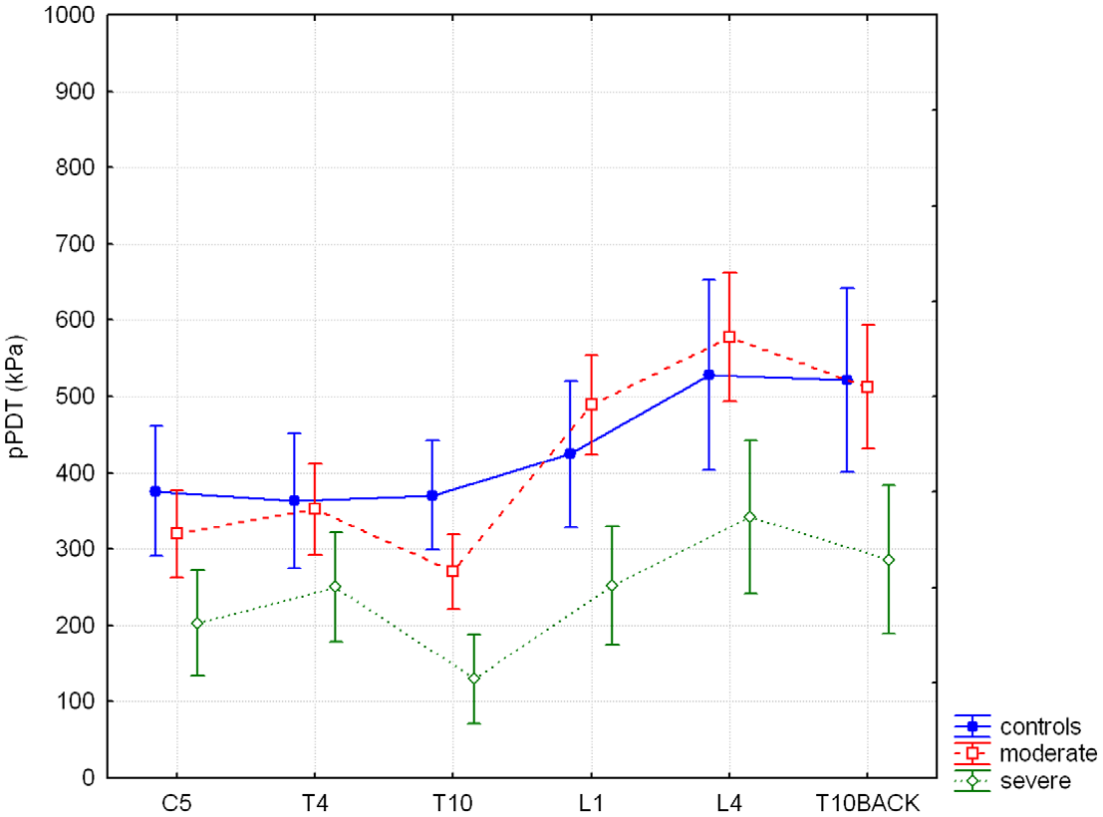
Pressure pain detection thresholds. The horizontal axis shows all six dermatomes, the vertical axis shows pressure pain detection thresholds (pPDT) in kPa. The difference between study groups and all six dermatomes for pPDT is significant (F = 3.75, p<0.0001). Results are means with 95% CI. ‘control’ is healthy control group, ‘moderate’ is moderate chronic pancreatitis group and ‘severe’ is severe chronic pancreatitis group.

For pPDT, there were significant differences between groups overall (GROUP; F = 8.88, p<0.001). Post-hoc analysis showed overall significantly lower thresholds for the severe chronic pancreatitis group compared to healthy controls (p = 0.001) and moderate pancreatitis group (p = <0.001). As expected, thresholds were significantly different according to dermatome of measurement (SITE; F = 45.28, p<0.0001). A significant interaction was found for SITE and GROUP (F = 3.75, p<0.0001) (Figure 2). Post-hoc analysis showed significantly lower thresholds for dermatome L1 (p = 0.04), L4 (p = 0.04) and T10BACK (p = 0.05) in the severe chronic pancreatitis group compared to the moderate chronic pancreatitis group.

**Figure 3:**
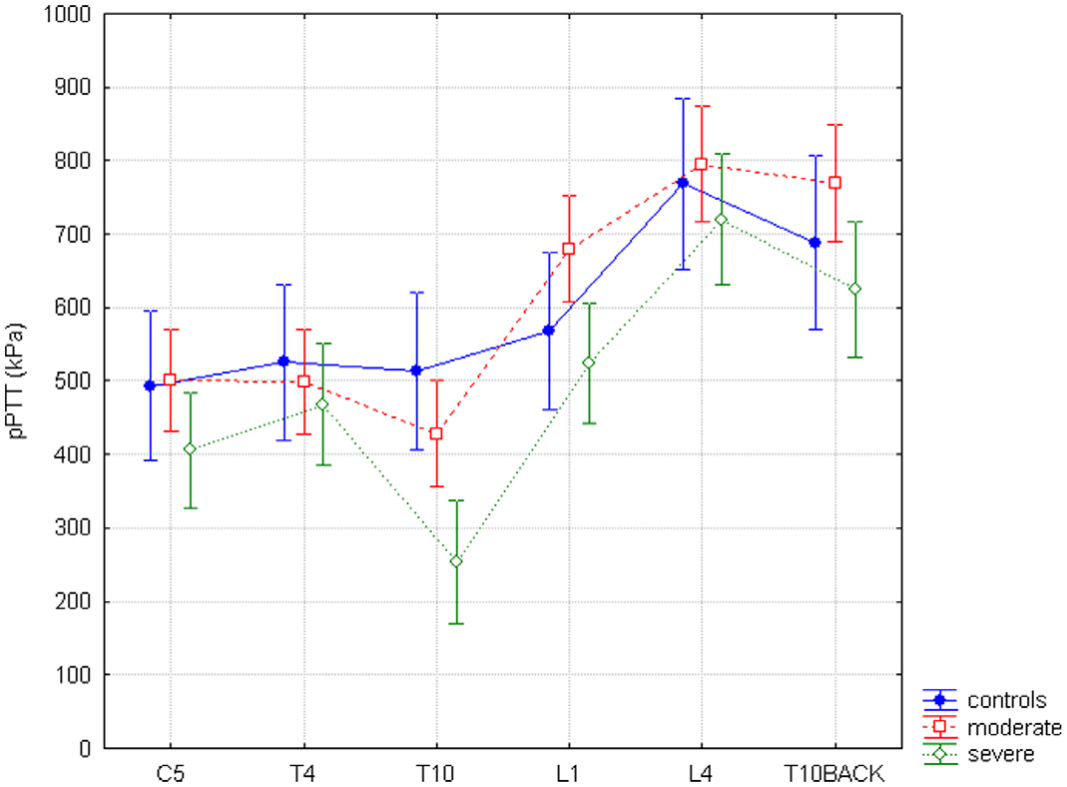
Pressure pain tolerance thresholds. The horizontal axis shows all six dermatomes, the vertical axis shows pressure pain tolerance thresholds (pPTT) mentioned in kPa. The difference between study groups and all six dermatomes for pPTT is significant (F = 3.27, p<0.001).

For pPTT there were no significant thresholds differences between groups overall, but only a trend (GROUP; F = 2.99, p = 0.06). Thresholds in the different dermatomes again significantly differed (SITE; F = 80.72, p<0.0001). A significant interaction between SITE and GROUP (F = 3.27 and p = <0.001) was seen (Figure 3). However post hoc analysis was not significant, with only a trend to lower thresholds between the severe pancreatitis group and healthy controls for the pancreatic dermatome (p = 0.07).

### Thresholds to Electrical Stimulation

**Figure 4:**
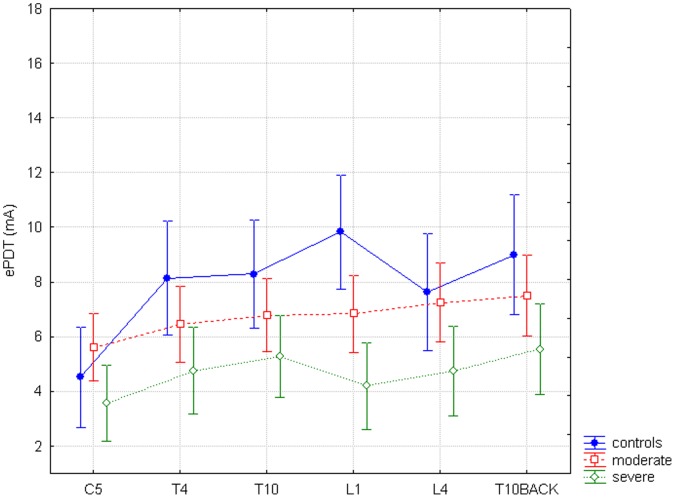
Electric pain detection thresholds. The horizontal axis shows all six dermatomes, the vertical axis shows electric pain detection thresholds (ePDT) in mA. The difference between study groups and all six dermatomes for ePDT is significant (F = 3.72, p<0.0001).

ePDT thresholds differed significantly according to dermatome (SITE; F = 17.48, p<0.0001) and groups overall (GROUP; F = 4.34, p = 0.02). Post hoc analysis for GROUP showed overall significantly lower thresholds for the severe chronic pancreatitis group compared to healthy controls (p = 0.007) and for the severe chronic pancreatitis group compared to the moderate chronic pancreatitis group (p = 0.03). There was also a significant interaction between SITE and GROUP (F = 3.72, p<0.0001) (Figure 4). Post hoc analysis was not significant, with only a trend to lower thresholds for the severe pancreatitis patients in L1 compared to healthy controls (p = 0.053), but without obvious difference between other dermatomes.

**Figure 5:**
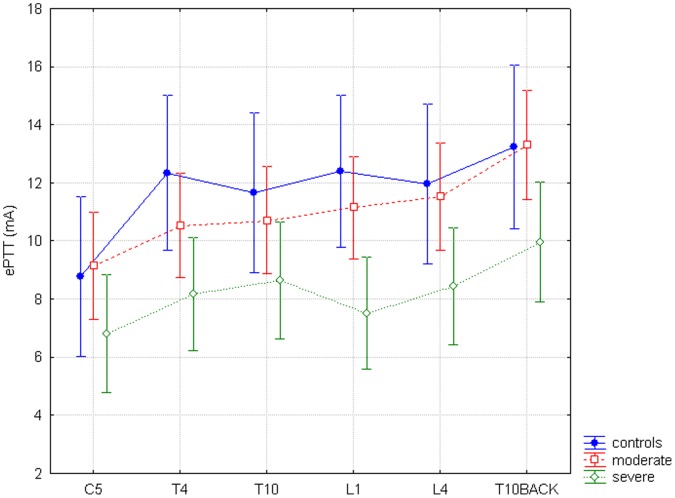
Electric pain tolerance thresholds. The horizontal axis shows all six dermatomes, the vertical axis shows electric pain tolerance thresholds (ePTT) in kPa. The difference between study groups and all six dermatomes for ePTT is non-significant (F = 0.91, p = 0.53).

For ePTT thresholds differed significantly for GROUP (F = 3.9, p = 0.03), with again a significant post-hoc analysis for overall lower thresholds for the severe chronic pancreatitis group compared to healthy controls (p = 0.02) and for the severe chronic pancreatitis group compared to the moderate chronic pancreatitis group (p = 0.02). Thresholds differed significantly for SITE (F = 12.98, p<0.0001). The interaction between SITE and GROUP was not significant (F = 0.91, p = 0.53) (Figure 5).

### Conditioned Pain Modulation

The baseline pressure pain tolerance thresholds for dermatome L4 were not significantly different between groups (pPTT L4: F = 0.8; p = 0.45). Chronic pancreatitis patients and healthy controls showed an increase in thresholds after the cold pressor task. The moderate chronic pancreatitis patients tolerated the cold pressor task for 66±59 sec and the severe chronic pancreatitis patients for 36±27 sec vs. healthy controls with 180±1 sec – which was significantly different overall (F = 52.2 and p<0.0001). Post hoc analysis was only significant for the healthy controls vs. the two pancreatitis groups (p<0.0001).

The effect of CPM was smaller in the patient groups compared to the controls, but the difference between groups was not significant (GROUP; F = 2.2; p = 0.13; controls: mean 32.8%±8.9% vs. moderate: 13.5%±21.4% vs. severe: 10.3%±39.9%). When all pancreatitis patients were taken together and compared to healthy controls, there was a significant difference (p = 0.04; controls: mean 32.8%±8.9% vs. pancreatitis: 12.0%±4.8%).

## Discussion

Our study confirms that patients with chronic pancreatitis show signs of central sensitization, manifest as lower pain thresholds compared to healthy controls. Our results suggest that patients with more severe disease exhibit more central sensitization. We were unable to demonstrate a relation between disease stage and effectiveness of inhibitory pain modulation.

### Altered Pain Processing

Peripheral nociception at the site of the pancreas spreads via ascending pathways of the spinal cord to supraspinal structures including the cortex. [28], [29] If neurons at the spinal cord undergo neuroplastic changes, these changes will typically manifest as segmental hyperalgesia in the corresponding segments. [11], [30] Increased nociceptive drive on secondary neurons leading to hyperexcitability and firing of supraspinal neurons at lower thresholds can then be expected to ultimately result in spreading and generalized hyperalgesia. [11] Our findings are compatible with the above described changes and EEG studies in chronic pancreatitis pain patients showing alterations in the organization of the pain matrix. [12], [31] The main interest of our study is its provision of first evidence that patterns of altered pain processing may also reflect disease stage and progression.

The effect of endogenous feedback systems on nociceptive input can be measured using the conditioned pain modulation (CPM) paradigm, which reflects effects of descending control from the brain on second-order neurons in the spinal cord. [32] Recent evidence suggests that patients with chronic painful diseases like chronic pancreatitis may exhibit less effective descending inhibitory control. [21], [33] Our study confirms this result.

Both peripheral and central pain signaling are potentially sensitized in chronic pancreatitis. Several studies provide evidence for peripheral visceral nerve damage in chronic pancreatitis. [11] The presence of central sensitization demonstrated in chronic pancreatitis patients in the present study (and others) means that pain signaling is exaggerated in the central nervous system too. This central sensitization can be due to either a direct increase of sensitivity of neural structures, or due to a loss of inhibitory modulation of neural structures. The latter is tested by the CPM paradigm and was unaffected by disease stage but greater in chronic pancreatitis patients vs. healthy controls. The lacking effect of disease stage on CPM could be real or due to lacking study power in the face of greater variability in CPM measures as compared to pain thresholds. [34] Clearly this topic needs further investigation in a larger patient collective.

### Clinical Implications

The findings concerning variations in supraspinal central sensitization in relation to disease stage suggest implications regarding treatment approaches in chronic pancreatitis. Firstly, it has been demonstrated that degree of hyperalgesia in neuropathic pain patients is inversely related to analgesic efficacy of opioids. [35] If this applies in chronic pancreatitis, then increasing hyperalgesia will be linked to decreasing analgesic efficacy of opioids – and probably also increased risk of opioid-induced hyperalgesia. [36] Secondly, therapeutic measures aimed at nociceptive deafferentation alone (e.g. splanchnic denervation, surgery aimed at specific anatomical causes, peripheral analgesia) are unlikely to be effective unless combined with specific treatments targeting central sensitization (e.g. gabapentinoids, tricyclic antidepressants and ketamine). [2], [7], [11], [37], [38] This is again more likely to be the case in patients with more hyperalgesia, which in turn appears related to disease stage. Finally, there are indications that long-term, ongoing nociceptive input may result in supraspinal alterations to pain processing becoming independent of peripheral nociceptive input (autonomy). [6] This likely renders therapeutic measures aimed at depressing peripheral nociceptive input ineffective, making treatment of altered central pain processing the foundation of pain treatment in these patients. [2], [11] Such a course of events leading to autonomy would appear more likely in patients with more hyperalgesia and a longer history of their disease – and may again be related to disease stage. The diagnostic and therapeutic implications of these observations deserve further study.

### Other Chronic Pain Disorders

Our results support the hypothesis that patients with more severe disease exhibit more central sensitization. This hypothesis is further supported by other studies regarding relationships between disease severity, degree of central sensitization and therapeutic effect. For CRPS, a clear relationship between degree of central sensitization and disease progression has been demonstrated. [39] In inflammatory bowel disease and endometriosis a relation has been found between segmental or generalized hyperalgesia as a sign of central sensitization and clinical difficulty in controlling pain. [16], [22], [40] Similar results have been described in the past in Crohn’s disease and ulcerative colitis, where the degree of central sensitization was related to extent of bowel inflammation. [41]–[44] Clearly more research is needed in chronic pain patients to establish the relation between disease severity, changes in the central nervous system processing and therapeutic success. As a result of this, recently we started a longitudinal observational study with serial QSTs in chronic pancreatitis patients who are early in their disease to describe changes in pain processing during disease progression.

### Methodological Considerations

A limitation of this study is the relatively small size of the two chronic pancreatitis disease stage subgroups. Nevertheless, even the present explorative study provides evidence of significant differences between healthy controls and a validated classification of pancreatitis disease stages. A better-powered study might have provided more robust and significant evidence across all the modalities and individual dermatomes we measured in our study.

Sensitization of neurons and the extent of sensitization could be different between different tissues (skin vs. deeper tissues) and dermatomes. This might explain the differences between electric pain thresholds (a more superficial stimulus) and pressure pain thresholds (a stimulus of deeper tissues) in different dermatomes.

The healthy control group is slightly younger than the pancreatitis group. However, the impact of aging on pain processing remains controversial, some studies described an increase of pain thresholds during aging [45], others showed no effect [46] and some showed a decrease in thresholds during aging [47].

A further important limitation of this study is that it is only cross-sectional. For definitive answers, a larger and longitudinal study will need to be performed. In this study only QST measurements were performed. Combining QST with EEG measurements or brain imaging would provide more detailed data on changes in the central nervous system in relation to pain processing on which to base more effective therapeutic strategies in the future. The M-ANNHEIM classification is at the moment the most comprehensive classification system for different stages of chronic pancreatitis and various degrees of clinical severity. In our study the classification proved simple, objective and accurate to apply [27].

### Conclusion and Summary

The present study confirms that chronic pancreatitis patients show signs of altered central processing of nociception compared to healthy controls. The study further suggests that these changes may be influenced by disease stage. These findings underline the need to take altered central pain processing into account when managing the pain of chronic pancreatitis and may have important implications for its treatment. More research is needed to further characterize the link between disease severity and progression and its relationship to altered pain processing and treatment in chronic pancreatitis and other chronic pain disorders.
